# DeORFanizing Candida albicans Genes using Coexpression

**DOI:** 10.1128/mSphere.01245-20

**Published:** 2021-01-20

**Authors:** Teresa R. O’Meara, Matthew J. O’Meara

**Affiliations:** a Department of Microbiology and Immunology, University of Michigan Medical School, Ann Arbor, Michigan, USA; b Department of Computational Medicine and Bioinformatics, University of Michigan, Ann Arbor, Michigan, USA; University of Georgia

**Keywords:** *Candida albicans*, coexpression, gene function

## Abstract

Functional characterization of open reading frames in nonmodel organisms, such as the common opportunistic fungal pathogen Candida albicans, can be labor-intensive. To meet this challenge, we built a comprehensive and unbiased coexpression network for C. albicans, which we call CalCEN, from data collected from 853 RNA sequencing runs from 18 large-scale studies deposited in the NCBI Sequence Read Archive. Retrospectively, CalCEN is highly predictive of known gene function annotations and can be synergistically combined with sequence similarity and interaction networks in Saccharomyces cerevisiae through orthology for additional accuracy in gene function prediction. To prospectively demonstrate the utility of the coexpression network in C. albicans, we predicted the function of underannotated open reading frames (ORFs) and identified *CCJ1* as a novel cell cycle regulator in C. albicans. This study provides a tool for future systems biology analyses of gene function in C. albicans. We provide a computational pipeline for building and analyzing the coexpression network and CalCEN itself at http://github.com/momeara/CalCEN.

**IMPORTANCE** Candida albicans is a common and deadly fungal pathogen of humans, yet the genome of this organism contains many genes of unknown function. By determining gene function, we can help identify essential genes, new virulence factors, or new regulators of drug resistance, and thereby give new targets for antifungal development. Here, we use information from large-scale RNA sequencing (RNAseq) studies and generate a C. albicans coexpression network (CalCEN) that is robust and able to predict gene function. We demonstrate the utility of this network in both retrospective and prospective testing and use CalCEN to predict a role for C4_06590W/*CCJ1* in cell cycle. This tool will allow for a better characterization of underannotated genes in pathogenic yeasts.

## Abstract (precis)

Candida albicans is a common and deadly fungal pathogen of humans, yet the genome of this organism contains many genes of unknown function. By determining gene function, we can help identify essential genes, new virulence factors, or new regulators of drug resistance, and thereby give new targets for antifungal development.

## INTRODUCTION

Coexpression analysis is based on the hypothesis that genes that are coordinately expressed under multiple diverse conditions and perturbations are likely to function in the same biological process (1). Coexpression networks are built from transcriptomic studies across a range of conditions, incorporating broad and unbiased analyses of gene expression at a global scale. The gene-by-condition matrix found in most transcriptomic studies can be used for differential gene expression analysis, where the effects of a perturbation or a mutation can be compared to a background or the wild type. In contrast, a coexpression network transforms the gene-by-condition matrix into a gene-by-gene matrix, or equivalently, a gene network, where the edge weight defines the degree of coexpression. To estimate this coexpression, several methods have been proposed, including the Pearson correlation coefficient, the Spearman correlation coefficient, or a partial correlation coefficient (2). Although coexpression approaches to identify gene function have been used extensively in the model yeast Saccharomyces cerevisiae and humans (1, 3, 4), it has only recently been applied to full effect in other fungi, as in the recent work from Meyer and colleagues (5, 6). Not only does coexpression have high predictive accuracy for gene function annotations, it also captures evolutionary-scale changes in cell identity (7). For nonmodel organisms such as Candida albicans, there are two important questions. (i) Is there utility in building a species-specific coexpression network, or are all the relevant coexpression signatures available through orthology with a model organism (e.g., S. cerevisiae)? (ii) How extensive does the transcriptional profiling need to be to generate useful coexpression networks? The second question is particularly relevant for emerging infectious diseases, as broad transcriptional profiling takes significant research investment to generate.

C. albicans is a common opportunistic pathogen of humans; it is both an asymptomatic colonizer of the mucosal surface of healthy individuals and a deadly invasive pathogen in immunocompromised patients. There is significant effort in determining gene function for C. albicans, with the goal of identifying essential genes, virulence factors, or regulators of drug resistance and thus expanding the target space for future antifungal development. Gene Ontology (GO) term annotation is one framework for describing gene function. For a gene to be fully annotated, it requires an understanding of the molecular function (MF), biological process (BP), and cellular component (CC). The C. albicans genome consists of 6,468 open reading frames (ORFs), and approximately 70% of these ORFs remain undercharacterized (8). As an extreme set, 1,801 ORFs have no biological process GO term annotation in the Candida Genome Database (9). In many cases, gene annotation information comes from inferred orthology with the model yeast S. cerevisiae, but these organisms last shared a common ancestor approximately 150 million years ago (MYA) (10), and even gene essentiality is not necessarily conserved across these species (8, 11). Current high-throughput approaches for gene function analysis include transposon insertion screens (11, 12), functional genomic screens of mutant libraries (8, 13–16), genetic interaction screens (17), yeast two-hybrid approaches (18), and other protein-protein interaction screens (19). However, there are also many transcriptomic analyses of C. albicans available through NCBI Sequence Read Archive (SRA), suggesting that it is now feasible to create a coexpression network for C. albicans as a complementary approach for predicting gene function.

Here, we generated a robust coexpression network from 853 sequencing runs from 18 available transcriptomic data sets for C. albicans, using rank correlation through the EGAD R package to build the network. We then added information from other modalities, including sequence similarity and S. cerevisiae protein-protein interactions from BIOGRID, as incorporation of orthogonal information allows for better gene function prediction (20). Retrospective analysis of the clustering identified high network connectivity of histone proteins and ribosomal proteins, validating the efficacy of this approach. We also demonstrate that there are distinct subnetworks for different glycosylphosphatidylinositol (GPI)-anchored cell wall proteins. We then applied this coexpression network to examining genes of unknown function in C. albicans and identified Ccj1 as a DnaJ-containing protein that acts as a regulator of cell cycle. This coexpression resource can be used by the research community for examination of gene function and network connectivity in C. albicans.

## RESULTS

### Coexpression networks in Candida albicans.

**FIG 1:**
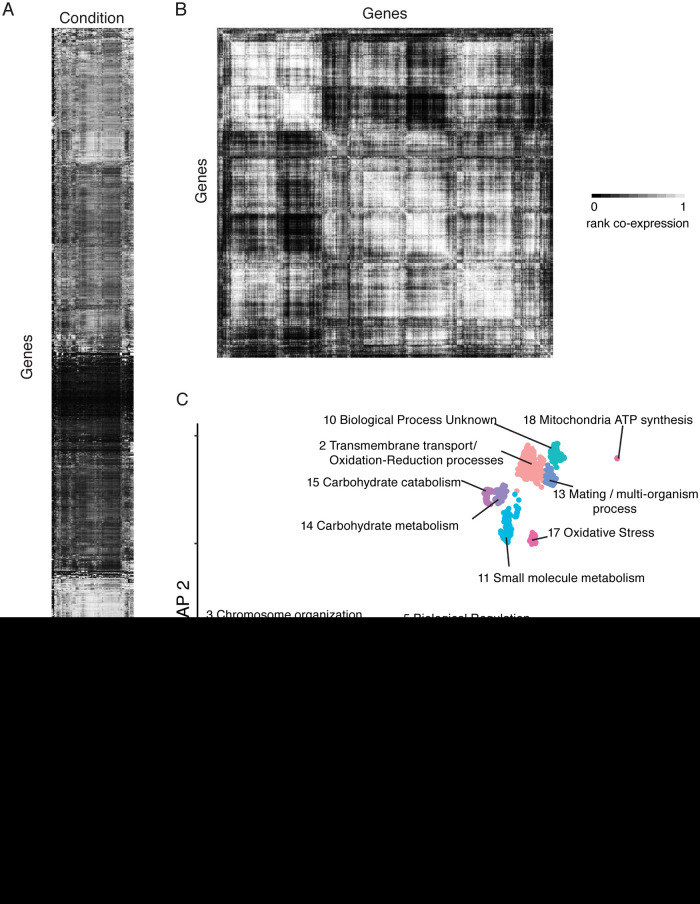
Generating a coexpression network for C. albicans. (A) A gene-by-environment heatmap generated from collected C. albicans RNAseq experiments from the SRA. The C. albicans genes are on the *y* axis, and conditions are on the *x* axis. (B) A gene-by-gene heatmap generated from Spearman rank correlation. (C) UMAP embedding reveals functional clusters. Annotations were determined by GO term enrichment of genes in each cluster.

A coexpression network is built through three stages: (i) collecting transcriptome data over multiple environmental conditions to generate a gene-by-condition expression matrix, (ii) measuring the correlation between all pairs of gene expression profiles to generate a gene-by-gene correlation matrix, and (iii) interpreting the correlation matrix as a network where the nodes represent genes and the edge weights represent the degree of coexpression. To build the Candida albicans coexpression network (CalCEN), we identified RNA sequencing (RNAseq) studies from the NCBI Sequence Read Archives (SRA), which we then filtered for studies with at least 20 C. albicans samples based on the guidelines from Ballouz and colleagues (2), yielding 12 unpaired and 6 paired end studies, listed in Table S1 in the supplemental material. By requiring at least 20 sequencing runs, this allows for measurement of both intra- and interstudy variation. The conditions for these experiments included differences in carbon source, coculture with host cells or bacteria, treatment with chemical perturbations, or differences in mutations, highlighting the diversity in conditions covered by these studies. Additionally, some of the experiments were performed on different strains of C. albicans, including SC5314, CAI-4, and DSY1050. To ensure that all studies were processed consistently, we collected the raw reads from SRA and realigned all of the data to the C. albicans SC5314 genome Assembly 22 coding transcripts using RSEM with bowtie2 (21–23) and generated a heatmap combining all of the RNAseq reads (Fig. 1A). One caveat is that some strains may have single nucleotide polymorphisms (SNPs) that would change the mapping of certain reads against the SC5314 reference genome. Since RNAseq of cocultured samples can lead to low coverage depth, we removed runs where greater than 50% of the genes had zero expression (see Fig. S1 in the supplemental material), yielding 853 runs in total. To control for the bias that more reads will map to longer genes, we used the fragments per kilobase of transcript per million mapped reads (FPKM) as the estimated expression for each gene under each condition. Because the primary aim of our study is to evaluate the utility of coexpression as a data source, we used the simple yet robust Spearman rank correlation to measure the correlation between gene expression profiles, as implemented in the EGAD R package (24) (Fig. 1B). Thus, for each pair of genes, we have a value between 0 and 1 representing the rank of coexpression among all pairs of genes.

10.1128/mSphere.01245-20.1 FIG S1 (A) A total of 1,399 RNAseq runs from 18 identified studies are scatter-plotted as the number of genes with nonzero expression versus the fraction transcripts that map exactly once. (B) The 853 runs that have nonzero expression for at least half of the genes (3,113), which are used to construct the CalCEN network, are shown in black. Download FIG S1, PDF file, 0.8 MB. Copyright © 2021 O’Meara and O’Meara. 2021 O’Meara and O’Meara. This content is distributed under the terms of the Creative Commons Attribution 4.0 International license.

10.1128/mSphere.01245-20.4 TABLE S1 RNA-Seq studies for Candida albicans coexpression analysis. Download Table S1, XLSX file, 0.01 MB. Copyright © 2021 O’Meara and O’Meara. 2021 O’Meara and O’Meara. This content is distributed under the terms of the Creative Commons Attribution 4.0 International license.

To visualize the coexpression network, we projected the network to two dimensions using UMAP (25), which aims to keep coexpressed genes closer together than noncoexpressed genes. Interestingly, we identify 18 distinct clusters (Table S2). Using Gene Set Enrichment Analysis (26) and GO term enrichment of the genes in each cluster, we can observe clear functional signatures (Fig. 1C), including cluster 9, which is enriched for cell cycle proteins, or cluster 18, which is enriched for proteins encoded on the mitochondrial genome.

10.1128/mSphere.01245-20.5 TABLE S2 Coexpression clusters from UMAP. Download Table S2, XLSX file, 1.3 MB. Copyright © 2021 O’Meara and O’Meara. 2021 O’Meara and O’Meara. This content is distributed under the terms of the Creative Commons Attribution 4.0 International license.

### Predicting gene function using multiple modalities.

**FIG 2:**
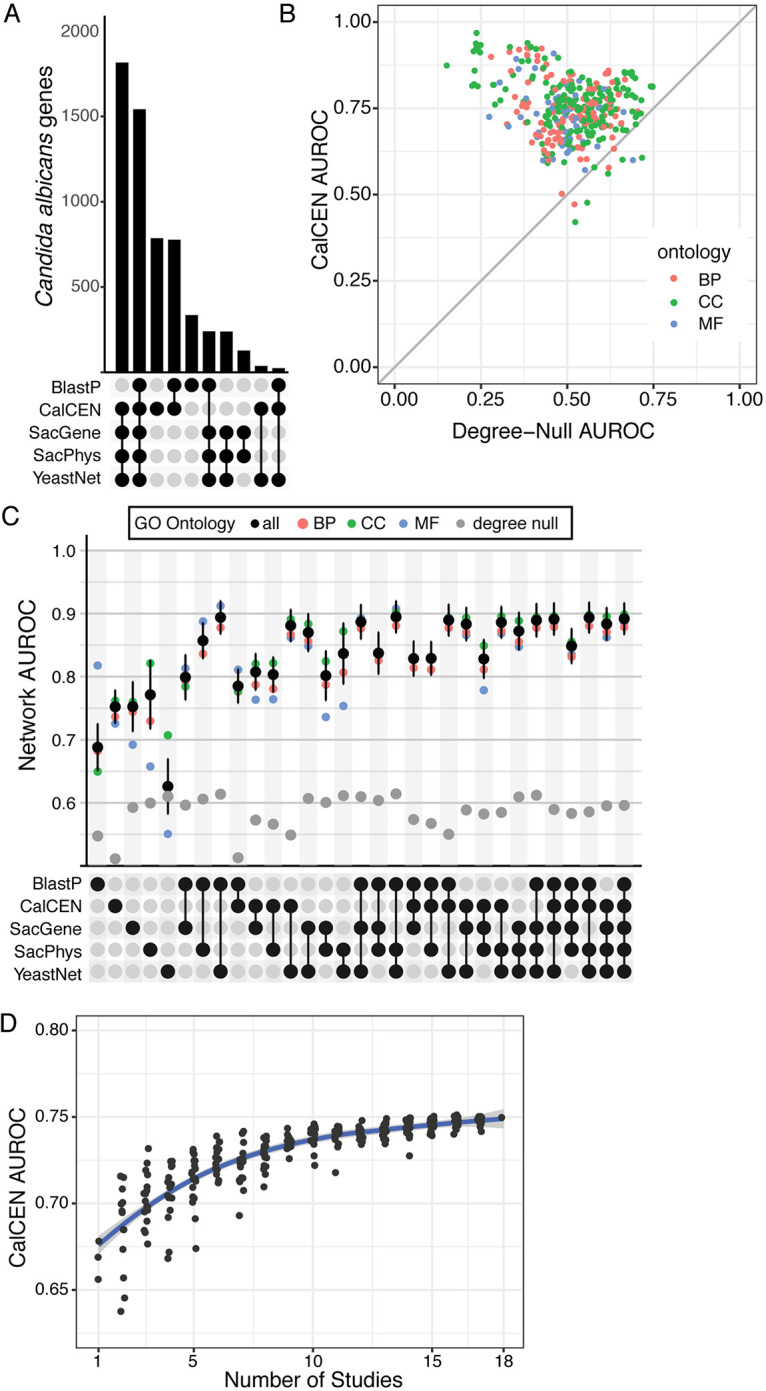
CalCEN provides a robust orthogonal approach to identifying gene function. (A) UpSet plot of network overlap. The BlastP network has a threshold at an E value of <1e−5, CalCEN has a threshold at the top 1%. Each bar in the upper region shows the number of gene nodes in the intersection of the set of networks indicated by the rows with filled circles in the lower region. (B) For each annotated GO term colored by ontology biological process (BP), cellular component (CC), or molecular function (MF), the CalCEN neighbor voting guilt by association (GBA) area under the ROC curve (AUROC) is plotted as a function of the degree-null (genes predicted based on their network degree) AUROC. (C) Mean neighbor voting GBA performance for individual and combined networks, indicted by the rows with filled circles in the lower region, for subontology terms (colored dots), all terms (black) with error bars representing the standard errors of the mean sover 10-fold cross validation replicas. Degree-null predictive accuracy for each network is shown in gray. (D) Mean neighbor voting GBA performance for the CalCEN built over random subsets of RNAseq studies. The blue curve represents a mean of a nonparametric locally estimated scatterplot scattering (LOESS) fit with standard deviation in dark gray. As the number of studies increases, the performance increases.

There are many networks that can be built for predicting gene function, each network can provide information about currently underannotated genes, and the different modalities can be combined for greater coverage and accuracy (20). To measure the information in CalCEN that is not already captured by other modalities, we compared the coexpression network to the BlastP, SacGene, SacPhys, and YeastNet genome-scale networks. The BlastP network was generated by comparing all pairs of proteins in C. albicans using Protein-Protein BLAST 2.2.30+ (27). We used the S. cerevisiae orthologs of C. albicans genes to build the SacGene and SacPhys networks using genetic and physical protein-protein interaction data collected from BioGRID (28, 29); similarly, we mapped the multimodal S. cerevisiae YeastNet v3 network to the C. albicans orthologous genes (30). For these analyses of orthology, we used the mappings from the Candida Genome Database, which takes in both sequence and synteny information (9). Filtering the coexpression network for the top 1% of coexpressed genes shows substantial coverage of the information captured in other networks, while also having information on over 550 genes that are not included in any other network (Fig. 2A).

To benchmark the utility of our coexpression network to predict gene function, we first performed a retrospective prediction of all of the GO term annotations collected from the Candida Genome Database and FungiDB (9, 31). Guilt by association (GBA) is a method to predict gene function by propagating annotation labels through a given network. We implement GBA prediction through neighbor voting, where the strength of a term predicted for a gene is determined by the fraction of neighbors in the network having the term. For each term, we constructed a ranked list of genes by the strength of the prediction and compared it to the currently known set of C. albicans GO term annotations using the area under the receiver operating characteristic curve (AUROC) score and averaging over a 10-fold cross validation. The receiver operating characteristic curve measures the trade-off between the true- and false-positive rates across different decision thresholds, and the AUROC is the integrated area under this curve. For this analysis, the true-positive results would be where the predicted GO term annotation matches the known GO term annotation collected from FungiDB and the Candida Genome Database. The AUROC is equivalent to the Mann-Whitney U test statistic, which is used in the Wilcoxon rank sum test. The AUROC takes values between zero and one, where a random predictor has 0.5 and a perfect predictor has 1.0.

We began by measuring the predictive accuracy of the network relative to uninformative baselines. The null baseline predictor that randomly predicts genes for each term yields an AUROC of on average 0.5. However, a stronger but also noninformative baseline for a network sorts the genes by their number of neighbors and predicts this single sorted list for every term. This degree null predictor is often a surprisingly strong predictor for retrospective gene function prediction because multifunctional genes, such as well-studied signaling hubs, tend to be both well connected and annotated for many functions. However, this predictor has low utility for prospective gene function prediction as it cannot find new genes for a function of interest, and it cannot find a new function for a gene of interest. If a network has a high degree null predictor relative to its neighbor voting predictor, it suggests that the neighbor voting predictor may be biased toward multifunctional genes (32, 33). The BlastP network, derived from whole gene sequence similarity (BlastP) has an average degree null AUROC of 0.55 ± 0.045 and network AUROC of 0.69 ± 0.037. Orthology networks from S. cerevisiae, derived from physical protein-protein interactions (SacPhys) or genetic interactions (SacGene) curated from BioGRID have average degree null AUROCs of 0.60 ± 0.042 and 0.59 ± 0.042 and average network AUROCs of 0.78 ± 0.054 and 0.75 ± 0.039. The integrated S. cerevisiae network (YeastNet) that incorporates literature coannotations has an average degree null AUROC of 0.61 ± 0.046 and an average network AUROC of 0.63 ± 0.044. We find that the CalCEN network has an average degree null AUROC of 0.51 ± 0.034 and a neighbor voting AUROC of 0.75 ± 0.026, indicating that the network has low multifunctionality bias relative to other networks (Fig. 2B and C and Fig. S2).

10.1128/mSphere.01245-20.2 FIG S2 For each annotated GO term colored by ontology biological process (BP), cellular component (CC), or molecular function (MF), the indicated network neighbor-voting guilt-by-association (GBA) area under the ROC curve (AUROC) is plotted as a function of the degree-null (genes predicted based on their network degree) AUROC. Download FIG S2, PDF file, 0.1 MB. Copyright © 2021 O’Meara and O’Meara. 2021 O’Meara and O’Meara. This content is distributed under the terms of the Creative Commons Attribution 4.0 International license.

When comparing the predictive accuracy of coexpression (Co-Exp) to the other networks, we see that the Co-Exp network has predictivity comparable to those of SacGene and SacPhys and significantly better than BlastP and YeastNet. When the networks are combined additively, we see that adding Co-Exp improves the predictive accuracy of each of the other networks, with Co-Exp and YeastNet being particularly predictive (Fig. 2C). Together, this demonstrates that we are capturing information with the CalCEN that is not included in previous networks and that addition of the CalCEN can improve gene function predictions.

A challenge in building the CalCEN was determining whether the number of RNAseq studies was sufficient to generate a robust network. To assess this, we asked how the predictive accuracy of the CalCEN changes based on the number of RNAseq studies used for generating the network. Embedding each RNAseq run based on the expression pattern for each gene (Fig. S3), we see that the runs cluster by study, suggesting that the between-study condition differences are greater than the within-study condition differences and that additional studies would increase coverage of the expression topology. To assess the impact of study diversity on retrospective gene function prediction, we sampled subsets of studies and recomputed the network and GBA performance. We found that the mean performance increased from ∼0.675 to 0.75 from 1 to 18 studies, with a minimum of 10 RNAseq studies needed for competitive performance AUROC compared with previous predictive methods. However, even at 18 studies, the performance of the coexpression network for GBA predictions has not yet saturated and may thus be improved when additional studies are added.

10.1128/mSphere.01245-20.3 FIG S3 The 853 RNAseq runs are embedded by reducing the 6,226 dimension gene expression profiles to 100 dimensions using principal component analysis and then to two dimensions using UMAP using min_dist = 0.5 and n_neighbors = 30. Runs were then labeled by their study accession and plotted using ggplot2. Download FIG S3, PDF file, 0.05 MB. Copyright © 2021 O’Meara and O’Meara. 2021 O’Meara and O’Meara. This content is distributed under the terms of the Creative Commons Attribution 4.0 International license.

### Retrospective identification of conserved gene clusters.

**FIG 3:**
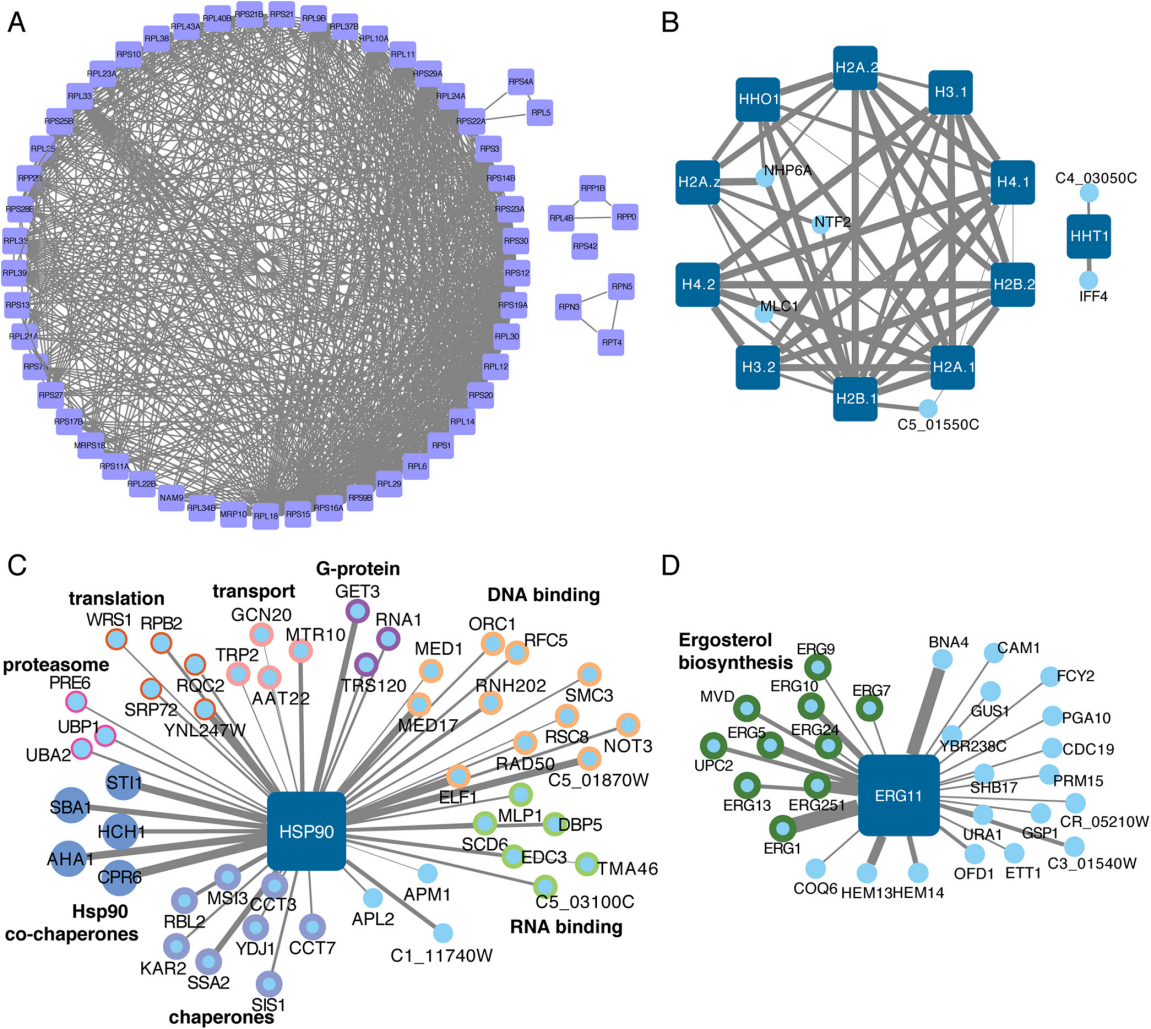
Retrospective analysis identifies functional clusters of genes in C. albicans. (A) Ribosomal proteins form a densely connected coexpression cluster. (B) Histone proteins are highly connected, except for the HHT1 variant histone protein. (C) Hsp90 is coexpressed with Hsp90 cochaperones, as well as multiple other functional classes of proteins. (D) Erg11 is coexpressed with other components of the ergosterol biosynthetic cascade. Nodes represent the genes, and the edge width corresponds to the degree of coexpression.

We then examined our coexpression network for its ability to identify specific gene clusters that have been previously identified in other organisms. Previous work on S. cerevisiae described a coexpression cluster of ribosomal proteins and other proteins involved in translation, as these proteins are coordinately regulated in response to nutrient conditions (1, 4). We used a set of known ribosomal proteins to seed the network, identified all of the first neighbors in the network, and then identified the coexpression edges for all of the genes in this set. This resulted in a densely connected cluster for many known ribosomal and proteasomal proteins in C. albicans (Fig. 3A). However, we also identified some known ribosomal proteins that were not contained within the main cluster, suggesting potentially differential regulation patterns that may relate to coexpression of subcomplexes. For example, Rpn3 and Rpn5 are colocated in the proteasomal lid, and Rpp0 and Rpp1B are both in the ribosomal stalk.

Another well-known cluster of proteins are the histone proteins, which are transcriptionally regulated with the cell cycle. In S. cerevisiae, this cluster is composed of 8 histone proteins (1), but in C. albicans, we observed that 10 histone proteins cluster with each other (Fig. 3B). Both H2A variant proteins (H2A.1 and H2A.2) are present in the C. albicans histone cluster (34); however, the H3 variant gene *H3.A/HHT1* is not connected with this cluster. This is consistent with the recent reports of a decreased abundance of *HHT1* compared with the canonical H3 proteins *HHT2* and *HHT21*, and the coexpression of *HHT1* with *IFF4* and *CPS1* is consistent with a potential transcriptional connection between *HHT1* and the biofilm circuit described by Rai et al. (35). In the main histone cluster, we also observed *NTF2*, a nuclear envelope protein, and *NHP6A*, a high-mobility group (HMG) protein that binds to and remodels nucleosomes, as connected with multiple histone proteins. Intriguingly, C5_01550C, a protein of unknown function, was also connected with both H2B.1 and H2A.1; however, this protein is also coexpressed with ribosomal proteins.

Hsp90 is a conserved and essential molecular chaperone that physically interacts with many C. albicans proteins to regulate their folding and function (19). The coexpression network for Hsp90 was able to identify five Hsp90 cochaperones (Fig. 3C) and seven additional chaperone proteins. However, it also identified clusters of genes involved in many core aspects of cell biology, including protein translation and degradation, consistent with the pleiotropic role of Hsp90 in the cell. In addition to proteins that act in a complex, we hypothesized that our network would identify genes that act in a single biosynthetic cascade. To test this, we examined our network for genes that coexpress with Erg11, the major target of the azole antifungals and part of the ergosterol biosynthetic cascade (36). By using just Erg11 as the seed, we identified eight additional genes in the ergosterol biosynthetic cascade that were coexpressed with Erg11 (Fig. 3D). Moreover, we identified Upc2, the transcription factor that regulates ergosterol biosynthesis, as part of this cluster. Notably, this approach also identified multiple genes involved in heme uptake, as well as others that are involved in general metabolism.

### GPI-anchored proteins form multiple, distinct coexpression clusters.

**FIG 4:**
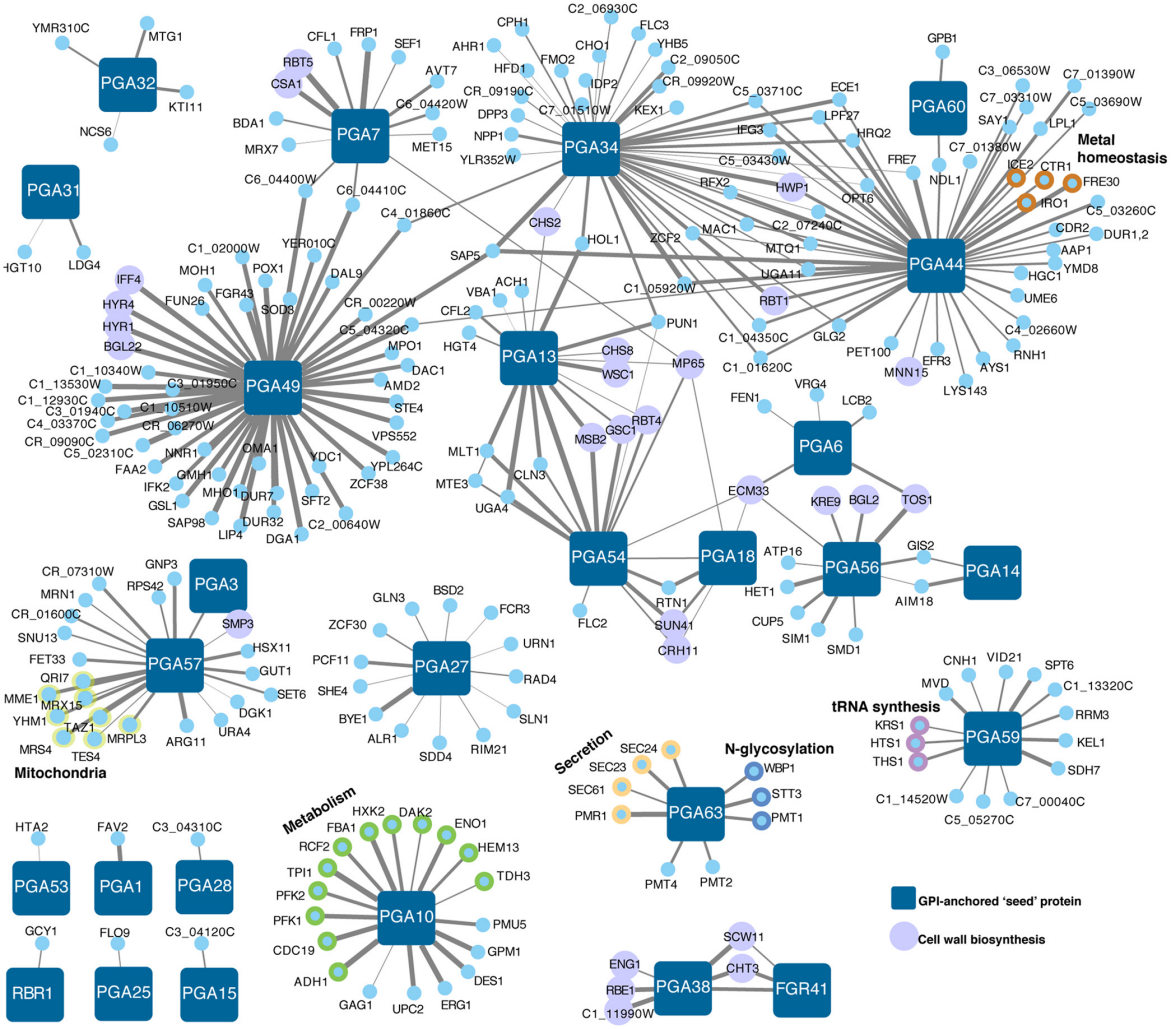
GPI-anchored proteins in C. albicans show distinct coexpression clusters. Twenty-seven GPI-anchored proteins were used as seeds to generate coexpression clusters. Genes were included as coexpressed if they passed the top 2% cutoff.

In C. albicans, the fungal cell wall plays an essential role in regulating interactions with host cells and tissues (8, 37, 38). The outer layer of the cell wall has many glycosylated proteins that are anchored into the cell wall via GPI motifs (39), and many of these proteins are not fully characterized. We selected a set of 27 GPI-anchored proteins based on predicted GPI anchor domains in the Candida Genome Database as seeds and examined their coexpression networks (Fig. 4A). This revealed that while there was a subset of GPI-anchored proteins that cluster with known cell wall biogenesis proteins, such as Pga38 and Pga54, there were also distinct and nonoverlapping coexpression networks that did not include cell wall-related processes. For example, Pga10 was most associated with proteins involved in metabolism, such as the Eno1 enolase, the Cdc19 pyruvate kinase, and the Pfk1 and Pfk2 phosphofructokinases, consistent with the role of Pga10 in using heme and hemoglobin as iron sources (40). Pga63, which is proposed to be a component of the COPII vesicle coat, is coexpressed with other secretion proteins (Sec61, Sec23, and Sec24), and proteins involved in N-glycosylation. Although COPII proteins are cytosolic, Pga63 was found to be on the plasma membrane in cell shaving experiments (41); therefore, future work is needed to demonstrate whether Pga63 is GPI anchored in C. albicans. Pga27, which is currently unannotated, is coexpressed with multiple transcription factors and the Rim21 and Sln1 signal transduction proteins that regulate cell wall remodeling in response to stress, which suggests that Pga27 may play a role in sensing or responding to stress. Similarly, the unannotated protein Pga59 is coexpressed with multiple proteins involved in RNA metabolism, including tRNA synthetases, while Pga57 is coexpressed with mitochondrial proteins. This highlights the potentially diverse biological functions of GPI-anchored proteins in C. albicans biology.

### Prospective testing of the coexpression network identifies a new role in cell cycle regulation for uncharacterized gene C4_06590W/*CCJ1*.

**FIG 5:**
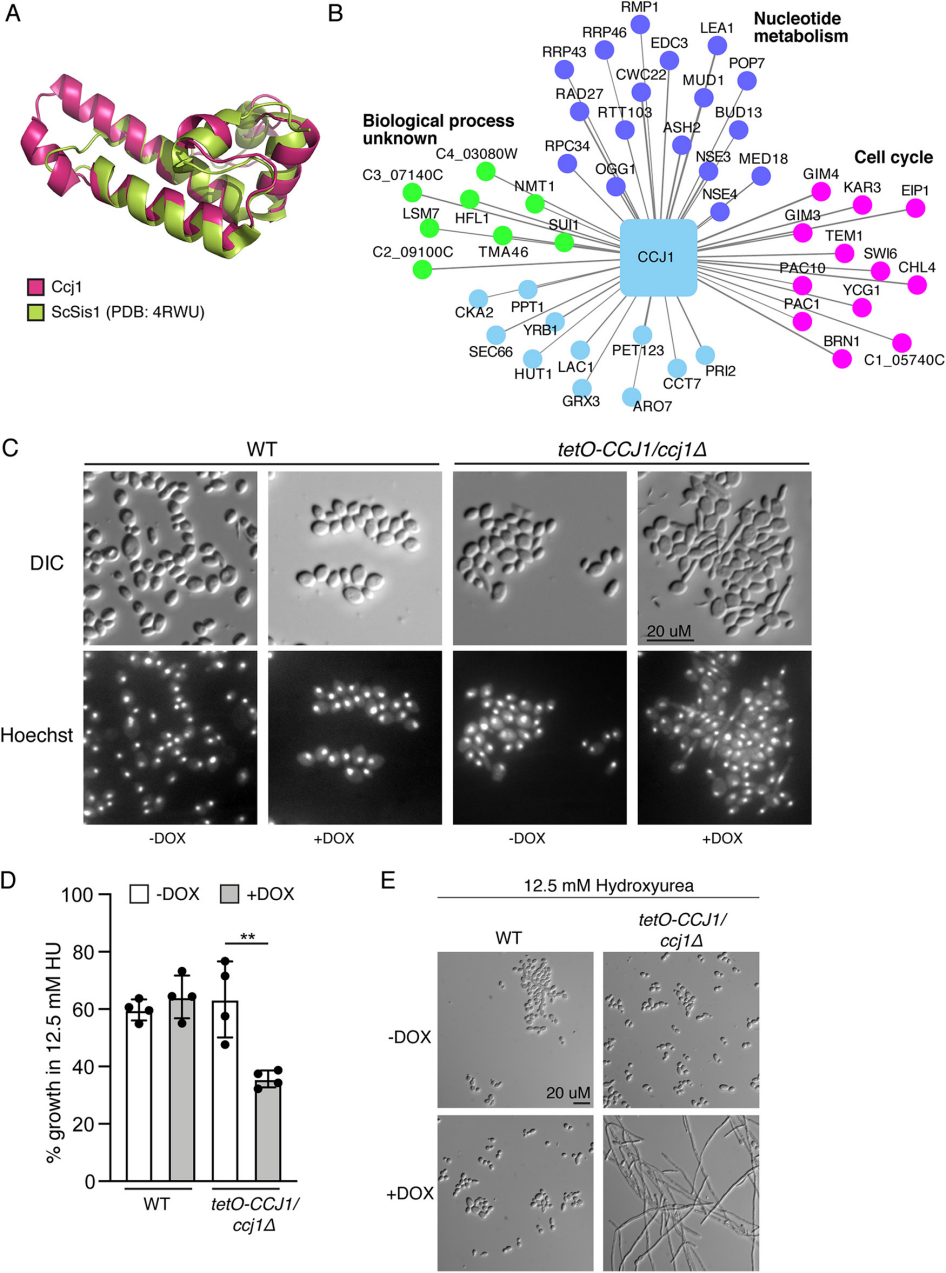
Identification of C4_06590W, or *CCJ1*, as a novel cell cycle regulator. (A) Ccj1 contains a J domain. TrRosetta was used to generate a *de novo* fold for the Ccj1 protein, and the structure was overlaid on the S. cerevisiae Sis1 (ScSis1) DnaJ protein crystal structure. (B) The coexpression network for Ccj1 identifies multiple cell cycle proteins. (C) Repression of *CCJ1* with 5 μg/ml doxycycline (DOX) results in aberrant filamentation in the absence of an inducing cue. Nuclei were stained using Hoechst. WT, wild type; DIC, doxycycline. (D) Repression of *CCJ1* with 5 μg/ml DOX results in hypersensitivity to 12.5 mM hydroxyurea (HU). Data are from two biological replicates, with two technical replicates each. Data were analyzed by *t* test; ** indicates *P* < 0.01. (E) Repression of Ccj1 results in hyperfilamentation in response to 12.5 mM hydroxyurea. Cells were incubated in YPD with 12.5 mM hydroxyurea and the presence or absence of 5 μg/ml of doxycycline overnight before imaging.

Many genes in the C. albicans genome do not have an assigned GO term annotation. The coexpression network can provide insight into genes without current annotation, suggesting functions that can then be tested experimentally. The gene C4_06590W is an uncharacterized protein that is present throughout the CTG clade and is not present in the model yeast S. cerevisiae; however, it has a similarly uncharacterized ortholog in Schizosaccharomyces pombe. The best homologous gene for C4_06590W in S. cerevisiae is the Sec63 protein, which contains both an N-terminal DnaJ domain and a C-terminal E-set domain. However, the presence of a conserved Sec63 ortholog in C. albicans (CR_04080C_A), and throughout the CTG clade, suggests that C4_06590W is not a Sec63 protein. To examine homology of the conserved DnaJ domain, we generated a model for the N terminus of the C4_06590W structure using TrRosetta (42), compared it with the available crystal structure of the S. cerevisiae Sis1 DnaJ-containing protein (43), and observed striking structural conservation of this DnaJ domain (Fig. 5A).

To investigate the function of C4_06590W, we examined the coexpression network using C4_06590W as the seed protein. This identified multiple genes involved in C. albicans cell cycle as clustering with C4_06590W (Fig. 5B), which led us to hypothesize that C4_06590W may be involved in cell cycle. We observed no overlap in the top 50 coexpressed genes when comparing CaSec63 as the seed protein to C4_06590W. In C. albicans, mutations in cell cycle often result in constitutive filamentation in the absence of an inducing cue (8, 44–47). Therefore, we examined the phenotype of the *tetO-C4_06590W/C4_06590W*Δ mutant strain in the presence or absence of doxycycline to repress target gene transcription (Fig. 5B). In this strain, one allele has been deleted, and the remaining allele is under the control of the *tetO* doxycycline-repressible promoter. When C4_06590W was repressed, the majority of the cells showed germ tube formation in the absence of an inducing cue (Fig. 5C). However, nuclear division appeared normal, with each cell containing just one nucleus (Fig. 5C). We then tested growth in the presence of hydroxyurea, a DNA synthesis inhibitor that induces S-phase arrest (47). Depletion of C4_06590W resulted in hypersensitivity to hydroxyurea, as observed by a decrease in growth (Fig. 5D) and a striking increase in the number of filaments at subinhibitory concentrations of the drug (Fig. 5E). Together, these data suggest that C4_06590W is involved in cell cycle control in C. albicans, and we have therefore proposed *CCJ1*, for cell cycle DnaJ, as the new gene name for C4_06590W.

## DISCUSSION

Coexpression has been shown to be a reliable means of identifying genes that share function in a variety of contexts. Genome scale coexpression networks built from bulk RNAseq studies have been used extensively for gene function prediction using guilt by association (1, 2, 48). Recently, coexpression networks using microarray data have been built for nonmodel organisms, including Aspergillus niger (5, 6). In A. niger, coexpression helped in the identification of a transcription factor network that controls the production of the A. niger antifungal peptide (AnAFP), as well as two novel transcription factors that regulate secondary metabolite clusters (6). Comparative coexpression between C. albicans and S. cerevisiae from microarray data helped identify regulatory motifs that were conserved between the two species (49). Here, we develop the CalCEN, a coexpression network for C. albicans from available large-scale, publicly available RNAseq studies. RNAseq has greater coverage and higher dynamic range than microarray studies, allowing for more nuanced analysis of coexpression patterns. We show that this network is built from sufficient data to meaningfully organize genes into functional modules and retrospectively predict gene annotations. We demonstrate the utility of this network to predict functional networks around several classes of genes, including ribosomal proteins and the ergosterol biosynthetic cascade. Moreover, the CalCEN analysis of histone proteins accurately identified a separation of the noncanonical Hht1 histone H3 protein (35) from the canonical histone protein complex, whereas both H2A variants (34) are highly coexpressed. Coexpression analysis of GPI-anchored proteins in C. albicans showed a variety of clusters with different enrichments for biological functions, highlighting that not all cell wall-associated proteins are involved in cell wall biosynthesis. Additionally, we used the CalCEN to identify a new regulator of cell cycle, *CCJ1*, which we verify contains a DnaJ domain using *de novo* structure prediction. Although the gene with the highest homology to *CCJ1* is *SEC63*, the C. albicans genome has a *SEC63* ortholog which has no overlap in coexpressed proteins with *CCJ1.* Many DnaJ proteins are cochaperones for the Hsp70 chaperone machinery (43), and Hsp70 proteins play important roles in regulating yeast cell cycle (50). Future work on Ccj1-interacting partners will be important for fully defining the mechanisms by which this protein contributes to cell cycle. The additional coexpression partners suggest that this may be through a role in nucleotide metabolism. Overall, we propose that the CalCEN will provide a method for predicting gene function for the C. albicans research community by providing testable hypotheses for experimentation. To assist the research community in using this network, we are integrating the CalCEN into the Candida Genome Database and FungiDB, as well as providing the network here (see Table S3 at https://figshare.com/articles/dataset/publication_data_CalCEN_v1_0_0_20201230_tar_gz/13515122). By integrating CalCEN into the databases, it will be possible to update the network when additional RNAseq studies are generated.

A limitation of this study is that we use simplistic methods for generating and analyzing the network. For example, we used Spearman rank correlation for building the network, we defined arbitrary thresholds when needed (such as the top 1% to compare network overlap), we combined networks by adding network weights, performed guilt by association using neighbor voting, and evaluated retrospective enrichment by using the area under the ROC curve. While more sophisticated methods may give higher predictive accuracy, an advantage of these simplistic methods is that they transparently show how the data lead to the predictions without complex global normalization. Further, the predictive accuracy of the simple methods gives a strong lower bound on the utility of the data. A future direction would be to integrate the CalCEN into multimodal gene function prediction methods that prioritize high accuracy over the top predictions (i.e., optimize the precision recall metric). This can be achieved, for example, through unsupervised network sparsification (51) or supervised machine learning for a given gene function prediction task (52). In demonstrating the utility of the CalCEN and making the methods and networks available through commonly used databases, we hope to enable their wider use in the fungal pathogenesis community, where the methods can be refined and integrated with additional data sources for gene function prediction.

## MATERIALS AND METHODS

### Network construction.

**(i) CalCEN.** RNA expression was estimated by aligning reads to Candida albicans SC5314, Assembly 22 (21) coding transcripts by converting from SRA to FASTQ format using fastq-dump from the NCBI SRA tools package and then aligned using the RSEM package v1.2.31 (22) with bowtie2 (23) using the default settings. Reads that did not map or mapped multiple times were discarded, and the percentages that mapped exactly once are shown in Fig. S1 in the supplemental material, plotted using the ggplot2 package (53) for R. The CalCEN network over genes was estimated by the Spearman rank correlation coefficients of the FPKM values across all runs using the EGAD R package (24).

**(ii) BlastP.** A sequence similarity network was created comparing all pairs of protein transcripts from C. albicans SC5314, Assembly 22 using protein-protein BLAST 2.2.30+ (27), yielding 103,400 associations. Protein-protein interaction networks were created from data collected from BioGRID build (3.4.161) (28), yielding 110,991 orthologous physical interactions between 3,953 C. albicans genes (SacPhys) and 395,437 orthologous genetic interactions between 3,942 C. albicans genes (SacGene). These protein-protein networks were then extended to include indirect associations with weights inverse of the shortest path (48). YeastNet v3, which is an integrative network for S. cerevisiae built from cocitation, coexpression, cooccurrence of protein domains, genomic context, genetic interactions, high- and low-throughput protein-protein interactions, phylogenetic profiles between yeast genes and three-dimensional (3D) structure of interacting orthologues (30). The overlap of the genes and interactions of these networks are shown in Fig. 2 using the UpSetR R package (54).

To assess the biological relevance of the coexpression network, functional annotations for curated by the Candida Genome Database (http://www.candidagenome.org/download/go/gene_association.cgd.gz) were collected on 12 June 2018, filtering for terms with qualifier != “NOT” and propagating up “is_a” and “part_of” term relationships using the GO.db R package (55). Terms were then filtered for those having at least 20 and at most 1,000 annotations, yielding 9,144 annotations for 169 biological process (BP) terms, 19,672 annotations for 215 cellular component (CC) terms, and 4,741 annotations for 85 molecular function (MF) terms. By evidence ∼73% annotations were inferred from electronic annotation (IEA), ∼11% were inferred from a mutant phenotype (IMP), ∼8% were inferred from direct assay (IDA), and less than 1% annotations were inferred for other evidence codes.

### Embedding of the Co-Exp network.

To embed the CalCEN genes, we used Monocle 3 (56) to preprocess the 6,226 × 853 expression matrix using principal-component analysis from 853 dimensions to 100 dimensions and then applied the UMAP algorithm to reduce the dimensions from 100 to 2. UMAP was used with parameters a = 50, b = 0.5, n_neighbors = 30, n_epochs = 2000, negative_sample_rate = 50, and repulsion_strength = 3, which builds on the R implementation of UMAP, uwot (53) (https://github.com/jlmelville/uwot), and the Approximate Nearest Neighbors Oh Yeah library (https://github.com/spotify/annoy). Clusters were identified using Leiden community detection algorithm (57) with parameters k = 30, num_iter = 10, and resolution = 0.1 and plotted with ggplot2 (53).

### Gene set construction.

A total of 6,468 C. albicans open reading frames were identified in the SC5314 Assembly 22 (21) database and downloaded from FungiDB (31). A total of 1,801 ORFs in this set did not contain a computed or curated GO term annotation for biological process. Twenty-seven GPI-anchored proteins were identified in the Candida Genome Database. Ribosomal proteins were identified by curated GO term annotation, and the first neighbors of the seed network were identified using the CalCEN.

### Protein structure prediction for CCJ1.

To predict the structure of the CCJ1 DnaJ domain, the sequence for residues 9 to 96 were submitted to the TrRosetta *de novo* structure prediction server (42), which built a deep multiple sequence alignment of 19,612 sequences and used a machine learning model to estimate the distances and relative angles of each pair of residues. These contact maps were then used to sample coarse-grain and full-atom protein folding conformation spaces to optimize a molecular mechanics force field to find low-energy conformations. The top resulting conformations were highly consistent with pairwise full-atom root mean square deviation (RMSD) of 0.15 Å, and when structurally aligned with 4RWU, a 1.25-Å X-ray crystal structure of the DnaJ-containing protein Sis1 from Saccharomyces cerevisiae, it has a full-atom RMSD of 1.6 Å (Fig. 5A), confirming the Interpro and Pfam sequence-based DnaJ domain annotation.

### CalCEN computational workflow.

Computational methods implemented here are available as an R package (github.com/momeara/CalCEN).

### Network visualization.

All network visualizations were generated in Cytoscape (58).

### Strains, reagents, and culture conditions.

All C. albicans strains were archived in 25% glycerol and stored at −80°C. Overnight cultures were grown in YPD (1% yeast extract, 2% Bacto peptone, 2% dextrose) at 30°C with rotation. The *tetO-C4_06590W/C4_06590W* strain and the associated parental wild-type strain used in this study were created as part of the GRACE tetracycline-repressible mutant collection (8, 59). Doxycycline (catalog no. MP219504410; Fisher) was dissolved in water and used at the indicated concentrations. To repress gene expression, overnight cultures of the relevant strains and controls were subcultured in the presence or absence of 5 μg/ml doxycycline (DOX) before use. Hydroxyurea (catalog no. A10831-06; Thermo Fisher) was dissolved in water and used at the indicated concentrations. Hoechst (catalog no. 15547; Cayman Chemical) was dissolved in water and used at 1 μg/ml.

### MIC assays.

Drug tolerance assays were performed in flat bottom 96-well plates (Alkali Scientific) using a modified broth microdilution protocol as previously described (19). The assays were performed in a total volume of 0.2 ml/well with twofold dilutions of the drug in YPD. Plates were incubated in the dark at 30°C for 24 h before reading optical density at 600 nm (OD_600_) values on a spectrophotometer (Synergy H1). Each strain was tested in technical and biological duplicate. Data at a single concentration of drug (the highest concentration where growth of the wild-type strain was not inhibited) are displayed.

### Microscopy.

To monitor C. albicans morphology, we used differential interference contrast (DIC) microscopy on an Olympus iX70 inverted microscope and a Hamamatsu FLASH4 complementary metal oxide semiconductor (CMOS) camera at ×40 magnification. For fluorescence microscopy, we used an X-cite series 120 light source with a 4′,6′-diamidino-2-phenylindole (DAPI) filter set. To monitor nuclei, cells were fixed and permeabilized with methanol before the addition of 1 μg/ml Hoechst stain and imaging. Representative images from two biological replicates are shown.
