# Supplementary figures and images for: DeORFanizing Candida albicans Genes using Coexpression
Source: mSphere. 2021 Jan 20;6(1):e01245-20. doi: 10.1128/mSphere.01245-20 (PMC7845621; doi:10.1128/mSphere.01245-20)

A

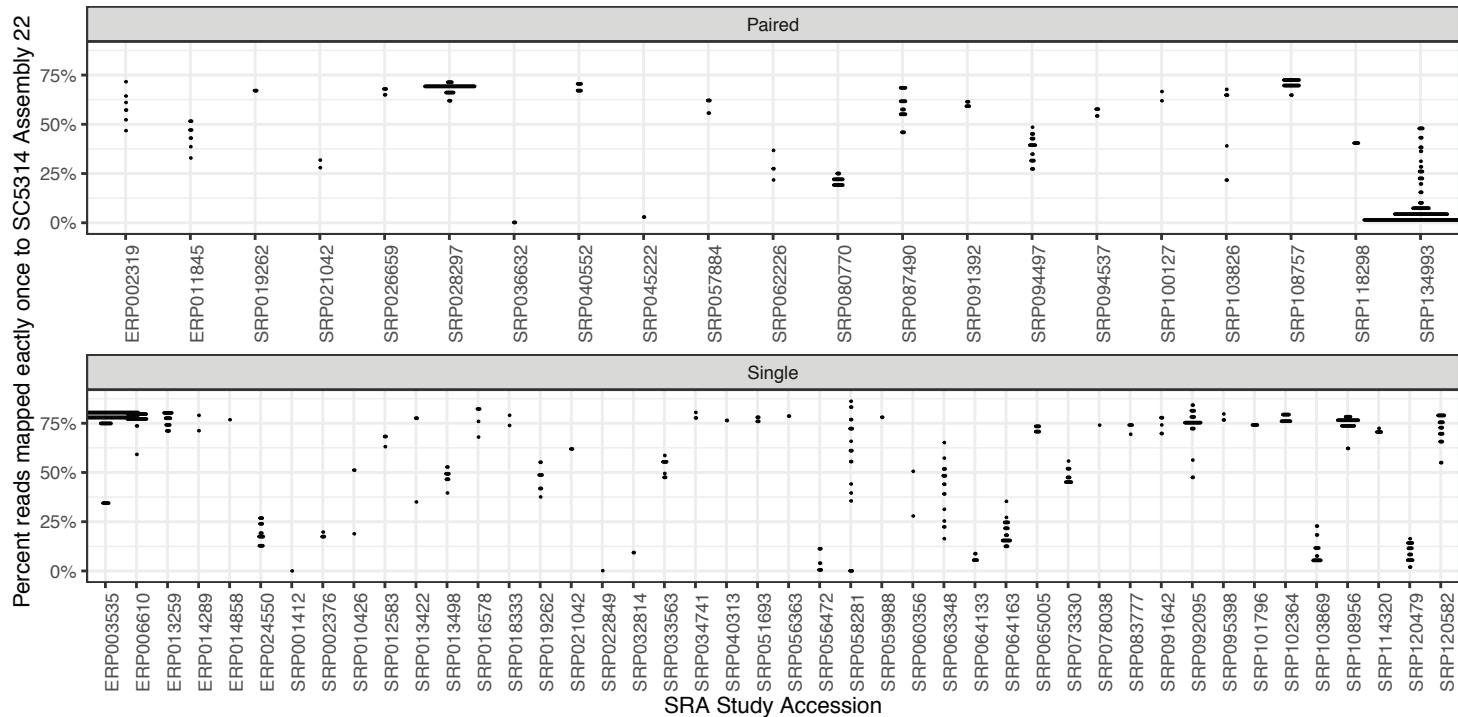

B

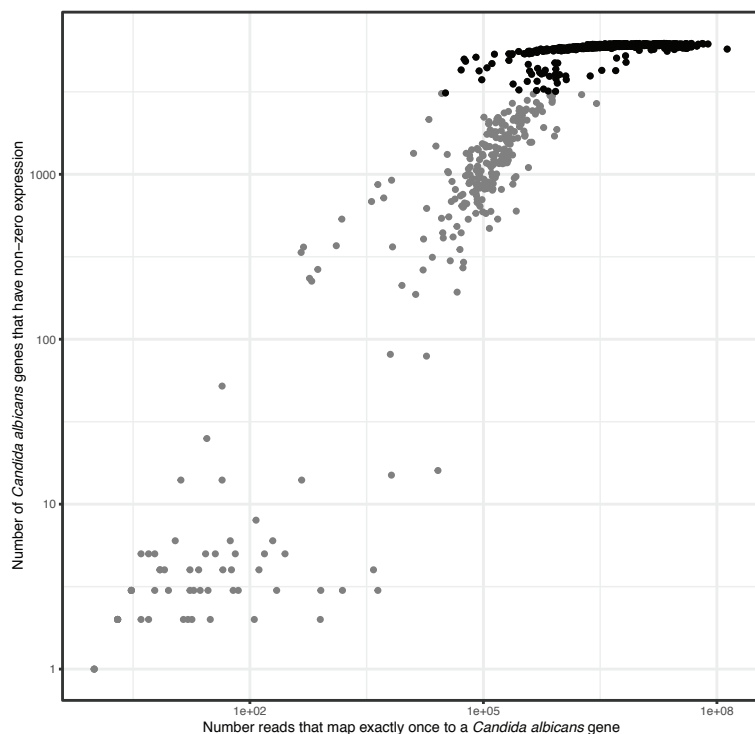

Supplement: FIG S1 [file mSphere.01245-20-sf001.pdf]

## GO term prediction

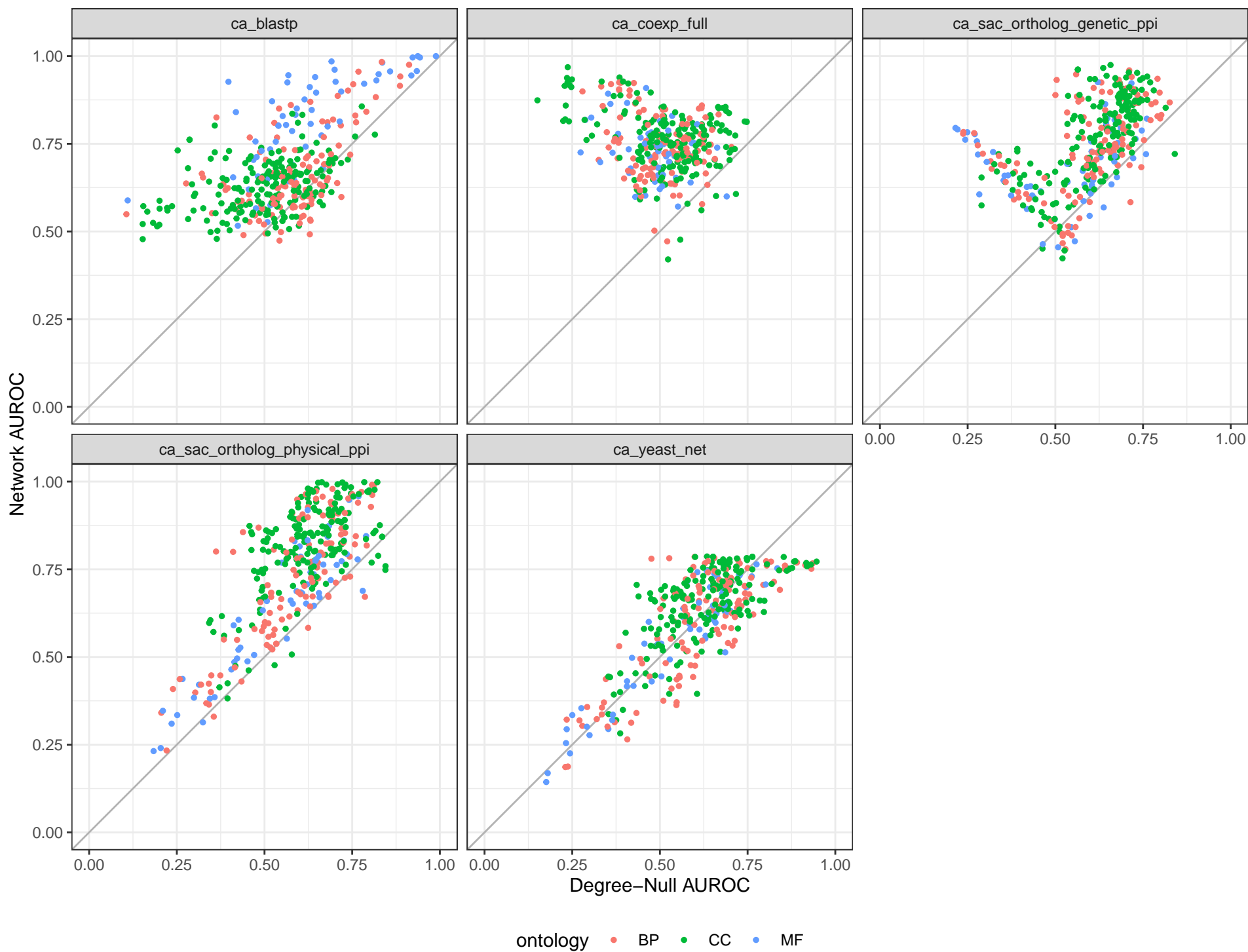

Supplement: FIG S2 [file mSphere.01245-20-sf002.pdf]

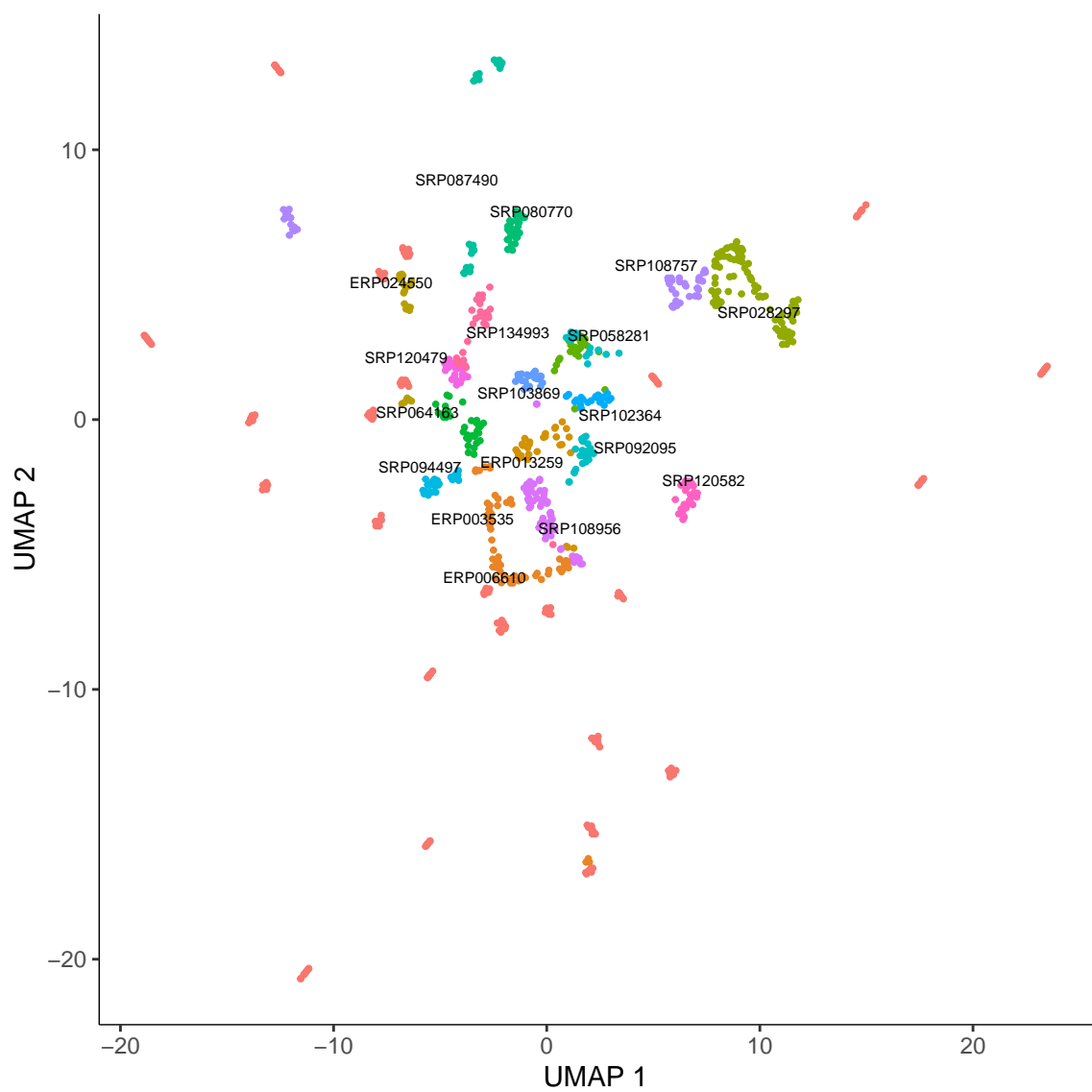

Supplement: FIG S3 [file mSphere.01245-20-sf003.pdf]
